# Functional Immunonutraceutical Supplementation Enhances Humoral and Innate Immune Dynamics in a Controlled Immune-Challenge Rabbit Model

**DOI:** 10.3390/nu18121872

**Published:** 2026-06-10

**Authors:** Ana Maria Plotuna, Ionela Hotea, Kalman Imre, Viorel Herman, Ileana Nichita, Alex-Cristian Moza, Ionela Popa, Cristian Zaha, Flavia Bochiș, Mihai Ioan Pop, Emil Tîrziu

**Affiliations:** 1 Department of Animal Nutrition, University of Life Sciences “King Mihai I” from Timișoara, 300645 Timișoara, Romania; anamaria.plotuna@usvt.ro; 2 Department of Food Safety and Hygiene, Faculty of Veterinary Medicine, University of Life Sciences “King Mihai I” from Timișoara, 300645 Timișoara, Romania; kalmanimre@usvt.ro; 3 Department of Infectious Diseases and Preventive Medicine, Faculty of Veterinary Medicine, University of Life Sciences “King Mihai I” from Timișoara, 300645 Timișoara, Romania; viorelherman@usvt.ro; 4 Department of Microbiology, Faculty of Veterinary Medicine, University of Life Sciences “King Mihai I” from Timișoara, 300645 Timișoara, Romania; ileananichita@usvt.ro (I.N.); alex.moza@usvt.ro (A.-C.M.); 5 Department of Semiology, Faculty of Veterinary Medicine, University of Life Sciences “King Mihai I” from Timișoara, 300645 Timișoara, Romania; ionela.popa@usvt.ro; 6 Horia Cernescu Research Unit, Faculty of Veterinary Medicine, University of Life Sciences “Regele Mihai I” from Timișoara, 300645 Timișoara, Romania; cristian.zaha@usvt.ro; 7 Department of Animal Resources Engineering, Faculty of Bioengineering of Animal Resources, University of Life Sciences “Regele Mihai I” from Timișoara, 300645 Timișoara, Romania; flavia.bochis@usvt.ro; 8 Department of Biotechnologies in Pharmaceutical Industry, Faculty of Biotechnologies, University of Agronomic Sciences and Veterinary Medicine of Bucharest (USAMV Bucharest), 59 Mărăști Blvd, Sector 1, 011464 Bucharest, Romania; mihai.pop24@bth.usamv.ro; 9 Department of Immunology, University of Life Sciences “King Mihai I” from Timișoara, 300645 Timișoara, Romania; emiltirziu@usvt.ro

**Keywords:** immunonutrition, donkey milk, bovine colostrum, royal jelly, lysozyme, immunoglobulins, immune–metabolic interactions, functional nutraceuticals

## Abstract

**Background/Objectives**: Immunonutrition uses dietary bioactive compounds to support immune function while preserving systemic physiological balance. Donkey milk, bovine colostrum, and royal jelly contain complementary antimicrobial, immunoglobulin-rich, and immunoregulatory components, but their combined effects remain insufficiently characterized. **Methods**: A 6-week controlled study was conducted in female rabbits assigned to four groups (*n* = 15/group): vaccinated only (G1), immunonutraceutical only (G2), vaccination plus immunonutraceutical (G3), and pre-conditioned immunonutraceutical followed by vaccination and continued supplementation (G4). Serum total immunoglobulins and lysozyme were measured longitudinally. Biochemical indices were monitored throughout the study, and hematological parameters were evaluated at the final time point. Mixed-effects models, generalized estimating equations, principal component analysis, and correlation-based systems analyses were applied. **Results**: Supplementation significantly modulated both humoral and innate immune responses. The strongest terminal immunoglobulin response was observed in G4 (26.00 ± 5.80 mg/mL), whereas sustained lysozyme elevation was most pronounced in supplemented groups, particularly G3 (3.13 ± 0.44 ng/mL). Within-subject analysis demonstrated significant innate–adaptive immune coherence (*p* = 0.000006). Biochemical analyses showed coordinated metabolic adaptation without evidence of organ toxicity, and hematological findings indicated preserved inflammatory and hematopoietic stability. **Conclusions**: Multi-component immunonutraceutical supplementation modulated humoral and innate immune dynamics in a timing-dependent manner while maintaining biochemical and hematological safety. These findings support the potential of combined donkey milk, bovine colostrum, and royal jelly as functional ingredients for coordinated immune support.

## 1. Introduction

Nutrition plays a central role in maintaining immune competence and systemic physiological homeostasis. Increasing attention has been directed toward the concept of immunonutrition, which describes the targeted use of dietary bioactive compounds capable of modulating immune function and inflammatory balance through nutritional pathways [1,2,3,4]. Dietary components may influence immune responses through multiple mechanisms, including regulation of mucosal barrier integrity, modulation of host–microbiota interactions, and metabolic signaling pathways linking nutrition and immune function [5,6,7]. Consequently, functional foods and nutraceuticals rich in antimicrobial proteins, immunoglobulins, and immunoregulatory lipids are increasingly investigated as nutritional strategies for supporting immune resilience and overall health [8,9,10].

Immunonutraceutical formulations may differ substantially according to the nature of their dominant bioactive compounds, including antimicrobial proteins, immunoglobulin-rich matrices, probiotics, prebiotics, bioactive peptides, and immunoregulatory lipids. In recent years studies have increasingly highlighted the potential of multi-component nutraceutical formulations capable of simultaneously targeting immune and metabolic pathways through complementary bioactive compounds. Such integrated approaches are particularly relevant under controlled immune-challenge conditions, where nutritional interventions may influence both innate and adaptive immune responses together with metabolic adaptation [3,5].

Among natural food matrices used in immunonutraceutical formulations, donkey milk, bovine colostrum, and royal jelly have attracted growing attention in nutritional science. Donkey milk possesses a distinctive whey protein profile and unusually high concentrations of antimicrobial enzymes, particularly lysozyme, which plays an important role in innate immune defense through its ability to hydrolyze bacterial cell walls and regulate microbial populations [11,12,13,14]. Beyond its antimicrobial activity, lysozyme has also been recognized as a multifunctional immune effector capable of modulating host–microbiota interactions and inflammatory signaling pathways [15,16]. These properties have led to increasing interest in donkey milk as a functional dairy product with potential benefits for immune health [17,18].

Another widely studied immunonutritional ingredient is bovine colostrum, the first milk produced after parturition. Colostrum contains high concentrations of immunoglobulins, growth factors, antimicrobial peptides, and cytokines that contribute to immune protection and gastrointestinal barrier integrity [19]. Several studies have reported that colostrum supplementation may influence immune responses and support mucosal immunity, primarily through gastrointestinal mechanisms involving gut-associated lymphoid tissue and host–microbiota interactions [18,20,21]. These gut-mediated effects may indirectly influence systemic immune competence and humoral responses.

A third component increasingly explored in functional nutrition is royal jelly, a secretion produced by worker bees that contains a variety of bioactive compounds including proteins, peptides, and unique fatty acids such as trans-10-hydroxy-2-decenoic acid (10-HDA). Experimental studies have demonstrated that royal jelly and its lipid components possess anti-inflammatory, antioxidant, and immunomodulatory properties capable of influencing immune signaling and cytokine production [22,23,24,25,26].

These ingredients have each been investigated individually for their potential immunological benefits; however, previous studies have largely focused on isolated immune markers rather than evaluating coordinated interactions between immune activation and metabolic adaptation [26]. Increasing evidence indicates that immune responses are closely linked to metabolic regulation, a concept known as immunometabolism, which describes how metabolic pathways influence immune cell activation and function [27,28,29,30,31].

Despite growing interest in functional immunonutrition, limited information is available regarding the combined use of donkey milk, bovine colostrum, and royal jelly within a standardized immunonutraceutical formulation. Furthermore, few studies have examined whether the timing of supplementation relative to antigen exposure influences coordinated innate and humoral immune responses. Animal models provide valuable tools for investigating such interactions under controlled experimental conditions. The rabbit (*Oryctolagus cuniculus*) is a well-established immunological model characterized by strong antibody responses and suitability for longitudinal blood sampling, allowing detailed monitoring of immune biomarker trajectories [32].

In this context, the present study aimed to evaluate the immunological and physiological effects of a multi-component immunonutraceutical composed of spray-dried donkey milk, bovine colostrum, and royal jelly administered to rabbits subjected to a controlled immune challenge. Specifically, we investigated whether supplementation could modulate systemic markers of innate immunity (serum lysozyme) and humoral immune responsiveness (total serum immunoglobulins), while simultaneously monitoring biochemical and hematological indicators of systemic health. We hypothesized that supplementation with this formulation would promote coordinated innate and humoral immune responses while maintaining metabolic and hematological stability.

## 2. Materials and Methods

### 2.1. Study Design and Ethical Approval

The study was conducted as a 6-week controlled, parallel-group experimental trial designed to evaluate the effects of a multi-component immunonutraceutical formulation (IMN) on biochemical, immunological and hematological parameters in rabbits. All procedures were performed in accordance with Directive 2010/63/EU of the European Parliament and of the Council on the protection of animals used for scientific purposes and Romanian Law No. 43/2014 [33,34]. The experimental protocol was approved by the Bioethics Committee of the University of Life Sciences “King Mihai I” from Timișoara, Romania (Approval No. 201/9 March 2023).

### 2.2. Animals, Housing, and Diet

Sixty clinically healthy female Pannon White rabbits (6 weeks of age) were obtained from a specialized commercial rabbit farm to ensure population uniformity in terms of sex, age, and body weight. The animal room was maintained under controlled environmental conditions, with an ambient temperature of 20 ± 2 °C, relative humidity of 60–65%, and a 12 h light/12 h dark photoperiod. Environmental parameters were monitored throughout the study and remained within the recommended ranges for rabbit husbandry [35]. Animals were housed individually in galvanized wire-mesh cages (44 × 26 × 30 cm), representative of commercial fattening systems [35]. The cages were elevated above ground level to allow fecal accumulation beneath the housing units, thereby maintaining hygienic conditions consistent with commercial rabbit production systems. Each cage was equipped with a feeder and automatic nipple drinker, and animals had ad libitum access to feed and fresh water. Rabbits were fed a standard commercial pelleted diet (GA Animal Nutrition SRL, Bacau, Romania; product code: GA600745). The analytical composition of the basal diet, as declared by the manufacturer, is presented in Table 1.

### 2.3. Experimental Design and Supplementation with Immunonutraceutical Formulation

All rabbits underwent a 1-week acclimatization period prior to the experiment to adapt to housing conditions, basal diet, and environmental factors. Animals were monitored daily to confirm normal health status and feed intake. After acclimatization, rabbits were randomly allocated into four experimental groups (*n* = 15 per group) using simple random assignment while maintaining overall homogeneity for age, sex, and body weight. No animals were excluded after allocation, and all animals completed the study and were included in the final analyses. Group G1 was vaccinated at baseline and received only the basal diet for 6 weeks. Group G2 received the dietary supplement for 6 weeks without vaccination. Group G3 was vaccinated at baseline and received the supplement throughout the 6-week period. Group G4 received the supplement for 3 weeks prior to vaccination, was vaccinated at week 3, and continued supplementation for an additional 3 weeks post-vaccination (Table 2). Vaccination was performed subcutaneously using a single 0.3 mL dose of Agalaxin^®^ (Romvac Company, Bucharest, Romania), an inactivated ovine vaccine containing Mycoplasma agalactiae (strain S/94). The vaccine was selected as a controlled heterologous antigenic stimulus to provide reproducible systemic immune activation without inducing clinical disease or severe inflammatory pathology [36]. The objective was to evaluate whether the immunonutraceutical formulation could modulate systemic humoral and innate immune responses under standardized antigenic challenge conditions while minimizing confounding effects associated with infectious disease models. Rabbits are widely used for evaluating vaccine immunogenicity and humoral immune responses under controlled experimental settings [36].

The immunonutraceutical formulation (IMN) produced by S.C. HYPERICUM IMPEX S.R.L. (Baia Sprie, Maramureș, Romania) consisted of 70% spray-dried donkey milk powder containing 1.5% lysozyme, 20% skimmed bovine colostrum containing 30% total immunoglobulins, and 10% lyophilized royal jelly standardized to 4% 10-hydroxy-2-decenoic acid (10-HDA). Spray-drying of donkey milk was performed at inlet air temperatures of approximately 160–180 °C and outlet temperatures of 70–90 °C, conditions that preserve functional dairy proteins while ensuring microbiological stability. Colostrum was mechanically skimmed by centrifugal separation to concentrate the protein fraction and reduce lipid content. Royal jelly was processed by lyophilization following freezing below −40 °C and dehydration under vacuum (<0.1 mbar), a technique known to preserve thermolabile bioactive compounds such as 10-HDA. Each daily dose contained 322 mg donkey milk powder, 92 mg colostrum powder, and 46 mg royal jelly powder. The composition values used in the formulation were provided by the manufacturer, and no independent post-spray-drying lysozyme activity assay was performed. The supplement was administered orally once daily using a needle-free syringe to ensure accurate dosing.

The experimental timeline was organized into weekly evaluation points from T0 to T5. T0 represented the baseline measurement prior to the initiation of dietary supplementation and/or vaccination. Subsequent time points (T1–T5) corresponded to weekly evaluations conducted throughout the six-week study period. At each weekly evaluation point (T0–T5), blood samples were collected for longitudinal assessment of immunological and biochemical parameters. Serum total immunoglobulins and lysozyme were measured at all time points to evaluate adaptive and innate immune dynamics, respectively. Biochemical analyses included liver-related, renal-related, lipid-related, and protein metabolism markers. Hematological parameters were evaluated only at the final time point (T5) to assess systemic physiological and inflammatory status following the intervention period. In groups receiving vaccination at baseline (G1 and G3), immunization was administered at T0. In the delayed-vaccination group (G4), rabbits received the immunonutraceutical supplementation during the first three weeks (T0–T3), followed by vaccination at T3, after which supplementation continued until the end of the experiment (T5).

### 2.4. Blood Sampling and Laboratory Analyses

Blood samples (approximately 2 mL) were collected weekly from the jugular vein using standard venipuncture technique as previously described for rabbits [37]. Samples intended for biochemical analysis were collected into plain tubes, allowed to clot at room temperature to permit serum separation, and centrifuged at 3000× *g* for 10 min. The serum was divided into two aliquots for biochemical analyses and immunological evaluation using ELISA kits. Samples were stored at 4 °C and analyzed within 24 h. Biochemical parameters, including glutamate pyruvate transaminase (GPT, U/L), glutamate oxaloacetate transaminase (GOT, U/L), total bilirubin (TBIL, mg/dL), creatinine (CRE, mg/dL), alkaline phosphatase (ALP, U/L), total cholesterol (TCHO, mg/dL), high-density lipoprotein cholesterol (HDL-C, mg/dL), triglycerides (TG, mg/dL), total protein (TP, g/dL), and albumin (ALB, g/dL), were determined using a FUJI DRI-CHEM automated analyzer (FUJIFILM, Japan), based on multilayer dry-slide reflectance photometry, according to the manufacturer’s instructions.

For hematological evaluation, blood samples were collected from the jugular vein, using the same venipuncture procedure described previously, into EDTA tubes and analyzed at the end of the experimental period by a certified diagnostic laboratory (Synevovet, Timis, Romania) using an automated hematology analyzer calibrated for rabbit blood. The evaluated parameters included white blood cell (WBC) count, red blood cell (RBC) count, hemoglobin concentration (HGB), hematocrit (HCT), mean corpuscular volume (MCV), mean corpuscular hemoglobin (MCH), mean corpuscular hemoglobin concentration (MCHC), red cell distribution width (RDW), platelet (PLT) count, mean platelet volume (MPV), reticulocyte count, and differential leukocyte counts, including neutrophils, lymphocytes, monocytes, eosinophils, and basophils.

### 2.5. Immunological Analyses

Serum total immunoglobulin concentrations were measured weekly using a rabbit ELISA kit (MyBioSource, San Diego, CA, USA; Cat. No. MBS731376) following the manufacturer’s protocol. Standards and serum samples were added to microplate wells pre-coated with specific capture antibodies and incubated to allow antigen–antibody binding. After washing to remove unbound components, an enzyme-conjugated detection reagent was applied, followed by substrate solution to generate a colorimetric reaction. The reaction was terminated using stop solution, and optical density (OD) was measured at 450 nm using a microplate reader. Concentrations were calculated from a standard calibration curve generated with the provided standards. All samples were analyzed in duplicate.

Serum lysozyme concentrations were determined using a rabbit ELISA kit (MyBioSource; Cat. No. MBS2602395), following the manufacturer’s protocol. After incubation of standards and samples in antibody-coated wells and completion of the washing steps, the enzymatic color reaction was developed and quantified by measuring optical density at 450 nm. Lysozyme concentrations were calculated using the corresponding standard curve. Samples were analyzed in duplicate.

### 2.6. Statistical Analysis

All statistical analyses and graphical visualizations were performed using Python (version 3.11; Python Software Foundation, Wilmington, DE, USA). Data are presented as mean ± SD for baseline comparisons and mean ± SEM for longitudinal analyses. Baseline differences among groups were evaluated using one-way ANOVA.

Longitudinal changes were analyzed using mixed-effects models and generalized estimating equations (GEE) to evaluate Group, Time, and Group × Time effects while accounting for repeated measurements and within-subject correlations across the experimental period. For hematological parameters measured at the final time point, normality and variance homogeneity were assessed, and group differences were analyzed using one-way ANOVA or Kruskal–Wallis tests, with false discovery rate (FDR) correction applied for multiple comparisons.

Associations between immune biomarkers and biochemical indices were evaluated using Spearman correlation analyses with within-subject centering, and area under the curve (AUC) values were calculated using the trapezoidal method. Principal component analysis (PCA) was performed as an exploratory multivariate approach to visualize biochemical variation across the study period using standardized variables.

Composite biochemical indices were derived from pooled z-score standardized biochemical variables to summarize liver, renal, lipid, and protein metabolism domains. An Overall Health Index was calculated by combining the individual domain indices. These indices were developed as exploratory measures of physiological adaptation and do not represent externally validated clinical scores.

Statistical significance was defined as *p* < 0.05 s. The sample size of 15 animals per group was selected based on previous nutritional and immunological studies in rabbits employing comparable experimental designs and longitudinal physiological monitoring together with practical and ethical considerations associated with repeated blood sampling and animal welfare and was sufficient to detect significant group × time interactions in longitudinal analyses [33,38,39].

## 3. Results

### 3.1. Total Serum Immunoglobulin (Ig)

**Figure 1:**
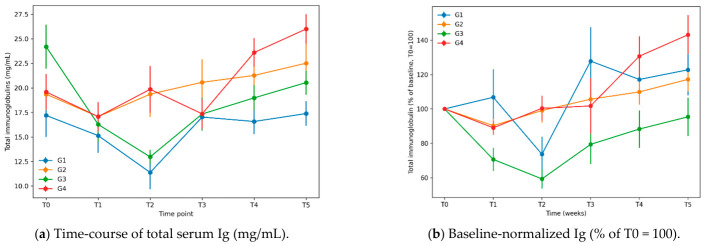
Longitudinal modulation of total serum immunoglobulins (Ig) across T0–T5. (**a**) Absolute concentrations (mg/mL); (**b**) relative change from baseline (T0 = 100%). Data are presented as mean ± SEM (*n* = 15 per group). Groups: G1 (V), G2 (IMN), G3 (V + IMN), G4 (IMN + V + IMN).

Total serum immunoglobulin (Ig) concentrations were measured longitudinally from T0 to T5 in all animals. At baseline, Ig concentrations did not differ significantly among groups (one-way ANOVA, F = 2.20, *p* = 0.0977), indicating comparable baseline humoral immune status. Baseline mean ± SD values (mg/mL) were 17.19 ± 8.46 (G1), 19.34 ± 6.25 (G2), 24.19 ± 8.72 (G3), and 19.59 ± 7.02 (G4) (Figure 1). Although G3 showed numerically higher Ig values, inter-individual variability was considerable and no statistically significant difference was detected. These findings confirm the absence of pre-existing humoral bias prior to intervention.

Longitudinal mixed-effects analysis revealed significant effects of Group (*p* < 0.05) and Time (*p* < 0.01), and a highly significant Group × Time interaction (*p* < 0.00001), indicating that Ig trajectories differed among treatment groups.

In G1, Ig concentrations decreased at T2 (11.38 ± 6.58 mg/mL), and subsequently increased toward baseline values by T3–T5. G2 demonstrated progressive elevation in Ig concentrations across time, reaching 22.51 ± 8.42 mg/mL at T5. This suggests that immunonutraceutical supplementation alone supports humoral immune activity independent of antigenic challenge. G3 exhibited a similar early decline to G1 but demonstrated improved stabilization at later time points. In contrast, G4 showed the most pronounced enhancement, achieving the highest terminal Ig concentration (26.00 ± 5.80 mg/mL at T5).

The significant Group × Time interaction indicates that immune responses depended on supplementation timing. The highest terminal immunoglobulin concentrations were observed in G4. However, interpretation of these differences should consider that vaccination was administered at different time points in G3 and G4, potentially influencing the observed humoral response trajectories. Supplementation alone (G2) also increased Ig levels, indicating timing-dependent modulation of humoral immunity.

### 3.2. Serum Lysozyme (LZM)

**Figure 2:**
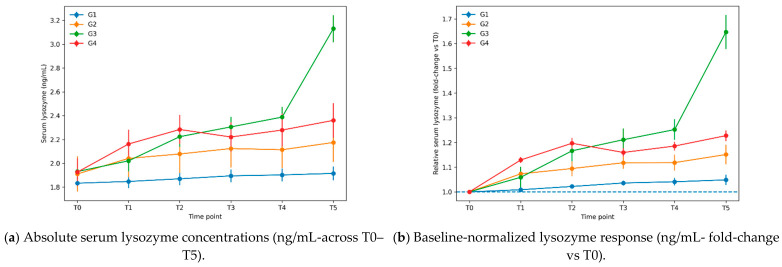
Serum Lysozyme: (**a**) absolute values (ng/mL); (**b**) fold-change relative to baseline (T0 = 1.0). Data are presented as mean ± SEM (*n* = 15 per group). Groups: G1 (V), G2 (IMN), G3 (V + IMN), G4 (IMN + V + IMN).

Baseline serum lysozyme concentrations did not differ among groups (*p* > 0.05), indicating comparable innate immune status prior to intervention. Baseline mean ± SD values (ng/mL) were 1.83 ± 0.22 (G1), 1.91 ± 0.57 (G2), 1.93 ± 0.31 (G3), and 1.92 ± 0.45 (G4) (Figure 2a,b).

GEE analysis showed significant effects of Time (*p* < 0.000001) and a strong Group × Time interaction (χ^2^ = 626.65, *p* < 0.000001; partial η^2^ = 0.652), indicating treatment-dependent lysozyme dynamics. Vaccination alone (G1) produced modest variation without sustained elevation, whereas supplementation (G2) induced progressive increases in lysozyme concentration, reaching 2.17 ± 0.63 ng/mL at T5. The combined treatment (G4) enhanced responses compared with vaccination alone (2.36 ± 0.56 ng/mL at T5), while G3 produced the strongest and most sustained lysozyme elevation, beginning before vaccination and persisting after antigen exposure (3.13 ± 0.44 ng/mL at T5).

To account for inter-individual variability, lysozyme concentrations were expressed as fold-change relative to baseline (T0). Baseline-normalized analysis revealed earlier and greater increases in supplemented groups, particularly G4, which exhibited elevated lysozyme prior to vaccination.

The significant Group × Time interaction (partial η^2^ = 0.652) indicates treatment-dependent differences in lysozyme trajectories, with higher and more sustained responses in the pre-conditioned group. Together with humoral findings, these results support coordinated modulation of innate and adaptive immune compartments, with particularly strong effects on innate immune activation.

### 3.3. Biochemical Profile

**Figure 3:**
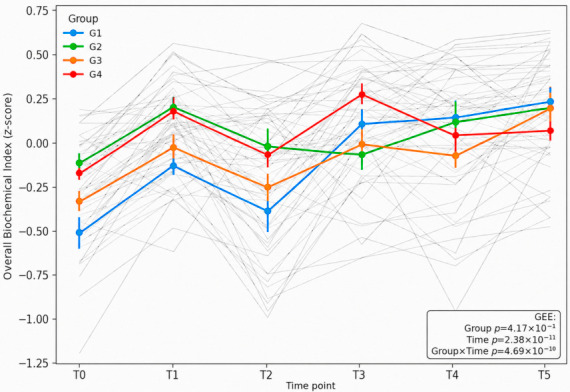
Composite Physiological Domain Index Time-course of Overall Biochemical Indices (z-scores) across T0–T5. Data are presented as mean ± SEM (*n* = 15 per group). Gray lines represent individual participants’ trajectories. Groups: G1 (V), G2 (IMN), G3 (V + IMN), G4 (IMN + V + IMN).

Standardized composite indices were calculated to summarize major physiological domains, including Liver (GPT, GOT, ALP, TBIL), Renal (CRE), Lipid (TCHO, HDLC, TG), Protein Metabolism (TP, ALB), and an Overall Biochemical Index integrating all markers (Figure 3). Although several biochemical variables demonstrated baseline variability among groups, most parameters remained within physiological ranges. Longitudinal mixed-effects and GEE analyses accounting for repeated measurements and within-subject temporal dynamics nevertheless revealed consistent treatment-related trends across the study period.

GEE analysis revealed significant effects of Group, Time, and Group × Time interaction (all *p* < 0.001) across biochemical indices. Liver and renal markers showed temporal modulation but remained within physiological ranges, indicating no hepatocellular injury or nephrotoxicity. The Lipid Health Index exhibited dynamic remodeling during immune challenge without dyslipidemia, while stable albumin levels indicated preserved protein metabolism and hepatic synthetic function. Serum amylase showed treatment-dependent changes but no persistent elevation suggestive of pancreatic stress. Overall, the biochemical profile reflected coordinated metabolic adaptation without evidence of systemic toxicity.

**Figure 4:**
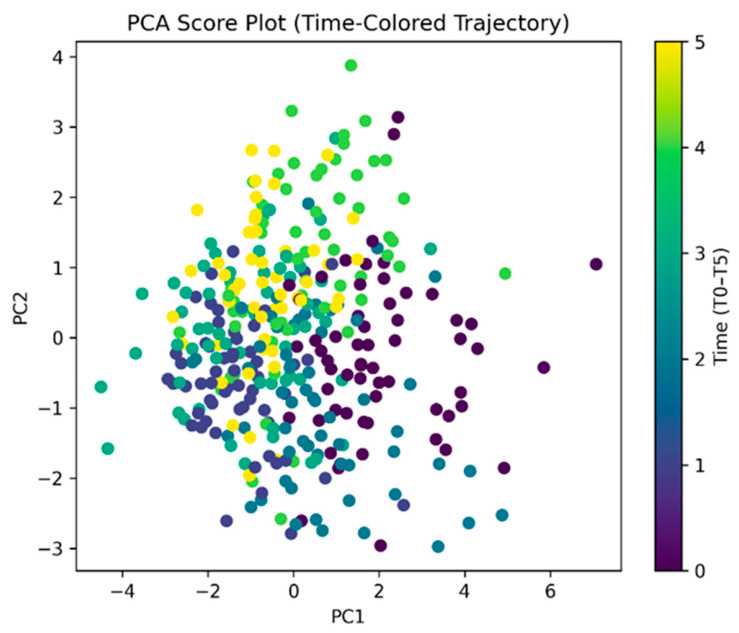
Principal Component Analysis (PCA) score plot (PC1 vs. PC2) colored by time (T0–T5).

Principal component analysis of standardized biochemical parameters identified PC1 (27.3% of total variance) and PC2 (15.6%) as dominant axes of metabolic variation (Figure 4). Because these two components together explained 42.9% of total variance, PCA was interpreted as an exploratory visualization of biochemical structure rather than definitive evidence of treatment-specific metabolic remodeling. The score plot suggested temporal organization of biochemical variation across the experimental period. Time-colored score plots demonstrated progressive displacement along PC1 from early (T0–T1) to later time points (T4–T5), indicating structured temporal metabolic adaptation. The loading structure of PC1 included contributions from hepatic enzymes, lipid parameters, and protein-related markers. No extreme clustering or directional divergence indicative of metabolic instability was observed.

Although significant modulation was observed across multiple domains, no biochemical parameters showed patterns consistent with hepatotoxicity, nephrotoxicity, pancreatic stress, dyslipidemia, or impaired protein synthesis. Overall, the results indicate adaptive immunometabolic remodeling rather than systemic organ dysfunction, demonstrating that the immunonutraceutical supports immune-related metabolic adaptation while maintaining biochemical safety.

### 3.4. Hematological Profile

Hematological analysis was performed at the final time point (T5) in all animals (*n* = 15 per group). Statistical evaluation was conducted using one-way ANOVA or Kruskal–Wallis tests depending on normality (Shapiro–Wilk) and homoscedasticity (Levene’s test) assumptions. False discovery rate (FDR) correction (Benjamini–Hochberg) was applied to account for multiple comparisons.

Significant group differences after FDR correction were observed for total leukocyte (WBC) count and red blood cell distribution width (RDW). WBC differed among groups (Kruskal–Wallis *p* = 0.00152; q = 0.01799; ε^2^ = 0.221), and RDW also showed significant variation (*p* = 0.00164; q = 0.01799; ε^2^ = 0.218). Other parameters, including lymphocytes, platelet (PLT) count, hematocrit (HCT), and mean corpuscular volume (MCV), showed nominal significance but were not significant after FDR correction, while neutrophils, neutrophil-to-lymphocyte ratio (NLR), hemoglobin (HGB), and erythrocyte (RBC) count did not differ among groups (*p* > 0.05).

Higher WBC values were primarily observed in G2, whereas G3 showed lower leukocyte counts despite exhibiting the strongest lysozyme response. These findings suggest that molecular immune biomarkers and circulating hematological parameters may reflect different aspects of immune regulation and should not necessarily be expected to vary in parallel. Absolute lymphocyte counts showed nominal differences (ANOVA *p* = 0.00657) that were not significant after correction. Although RDW remained significant, RBC, HGB, and HCT were stable, and erythrocyte indices remained within physiological ranges, indicating preserved oxygen transport and no evidence of anemia. Platelet differences were not significant after FDR correction and were not associated with neutrophilia or elevated NLR.

Overall, the hematological profile suggests selective immune-related modulation without systemic inflammation or hematological toxicity. The moderate WBC increase likely reflects enhanced immune readiness within physiological limits.

### 3.5. Integrated Correlation and Systems Analyses

#### 3.5.1. Innate–Adaptive Immune Coherence

To investigate whether humoral and innate immune responses were dynamically coordinated, correlation analyses were performed between total serum immunoglobulin concentration (Ig) and serum lysozyme (LZM) across all time points (T0–T5).

**Figure 5:**
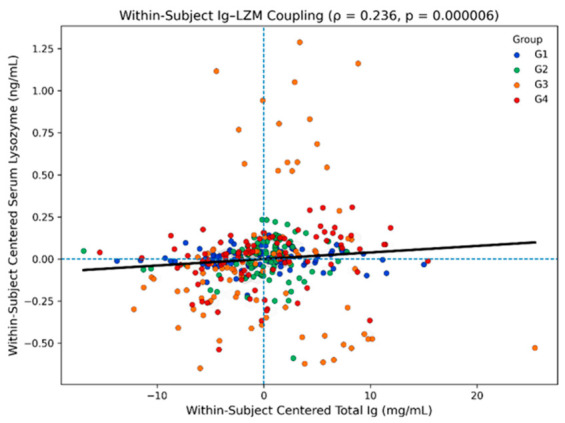
Within-subject centred analysis revealed significant innate–adaptive immune coherence Each point represents deviations from an individual animal’s mean across T0–T5. Coloured points indicate experimental groups G1 (V), G2 (IMN), G3 (V + IMN), G4 (IMN + V + IMN). The solid line represents the fitted regression model.

In the pooled dataset (*n* = 360), a weak but significant positive correlation was observed (Spearman ρ = 0.106, *p* = 0.043984). To reduce the influence of baseline differences between animals, a within-subject centered analysis was performed by subtracting each animal’s mean across time points (Figure 5). This analysis revealed a stronger association (Spearman ρ = 0.236, *p* = 0.000006; q = 0.000077), indicating that increases in humoral immunity were accompanied by parallel increases in innate immune activity within the same animal, reflecting coordinated immune modulation.

Within-subject centred analysis revealed a modest but statistically significant positive association between serum immunoglobulin and lysozyme dynamics, suggesting coordinated temporal variation between humoral and innate immune biomarkers. These findings establish that the IMN modulates immune integration rather than merely increasing individual biomarker levels.

#### 3.5.2. Immune–Biochemical Coupling

**Figure 6:**
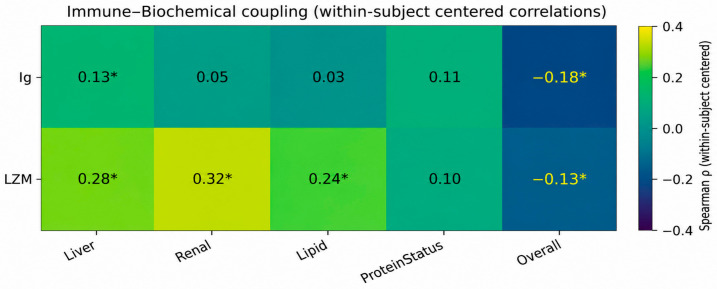
Immune–Biochemical Correlation Heatmap. Color intensity reflects the magnitude and direction of Spearman’s ρ values. Asterisks indicate statistically significant associations after false discovery rate correction (FDR–adjusted q < 0.05). Lysozyme demonstrates coordinated coupling with liver, lipid, and overall metabolic domains.

To determine whether immune modulation occurred in parallel with metabolic adaptation, within-subject centred Spearman correlation analyses were performed between immune biomarkers (Ig and LZM) and composite biochemical domain indices (Liver, Renal, Lipid, Protein Metabolism, and Overall Biochemical Index) across all time points (T0–T5; *n* = 360 observations). Within-subject centring was applied to remove inter-individual baseline differences and isolate dynamic covariation (Figure 6).

Lysozyme (LZM) showed significant positive correlations with the Liver Index (ρ = 0.319, *p* = 6.20 × 10^−10^, q = 6.20 × 10^−9^), Lipid Index (ρ = 0.277, *p* = 8.84 × 10^−8^, q = 4.42 × 10^−7^), and Overall Biochemical Index (ρ = 0.242, *p* = 3.38 × 10^−6^, q = 1.13 × 10^−5^), and a smaller inverse association with the Renal Index (ρ = −0.134, *p* = 0.0112, q = 0.0224). Immunoglobulin (Ig) showed weaker but significant associations with the Renal Index (ρ = −0.177, *p* = 7.24 × 10^−4^, q = 1.81 × 10^−3^) and Lipid Index (ρ = 0.129, *p* = 0.0142, q = 0.0237), while correlations with the Liver and Overall Indices were not significant after FDR correction. Overall, lysozyme showed stronger coupling with metabolic remodeling, whereas Ig exhibited more modest associations.

This analysis shows that immune activation was metabolically integrated rather than isolated. Lysozyme appeared to be a key immunometabolic marker, linking innate immune activity with liver and lipid metabolic changes. These results suggest that the immunonutraceutical promotes coordinated immune–metabolic regulation without signs of biochemical toxicity.

#### 3.5.3. Immune–Hematology Coupling

To assess whether molecular immune responses were reflected in circulating immune cells, correlations were performed between immune activity and hematological parameters measured at the final time point (T5). Area under the curve (AUC) values for immunoglobulin (Ig) and lysozyme (LZM) were calculated across T0–T5 using trapezoidal integration (*n* = 60) to represent cumulative immune responses. These values were correlated with WBC, lymphocytes, neutrophils, NLR, RDW, and platelet (PLT) count.

No significant associations were detected between Ig AUC and WBC (ρ = −0.010, *p* = 0.937), lymphocytes (ρ = −0.079, *p* = 0.550), neutrophils (ρ = 0.021, *p* > 0.80), or NLR (ρ = −0.139, *p* = 0.290). Similarly, LZM AUC did not correlate significantly with WBC (ρ = 0.091, *p* = 0.489), neutrophils (ρ = −0.032, *p* > 0.75), or NLR (ρ = −0.084, *p* = 0.524). A modest positive trend was observed between LZM AUC and lymphocyte count (ρ = 0.221, *p* = 0.089), although this did not reach statistical significance. No significant relationships were identified between immune AUC values and erythrocyte indices or platelet count after correction for multiple testing.

Correlation analysis between integrated immune responses (AUC) and hematological parameters at T5 showed no significant associations between Ig AUC or LZM AUC and leukocyte indices. These findings indicate preserved hematological homeostasis, suggesting that enhanced humoral and innate responses were not accompanied by changes in leukocyte distribution or inflammatory balance.

#### 3.5.4. Systems Integration Network

**Figure 7:**
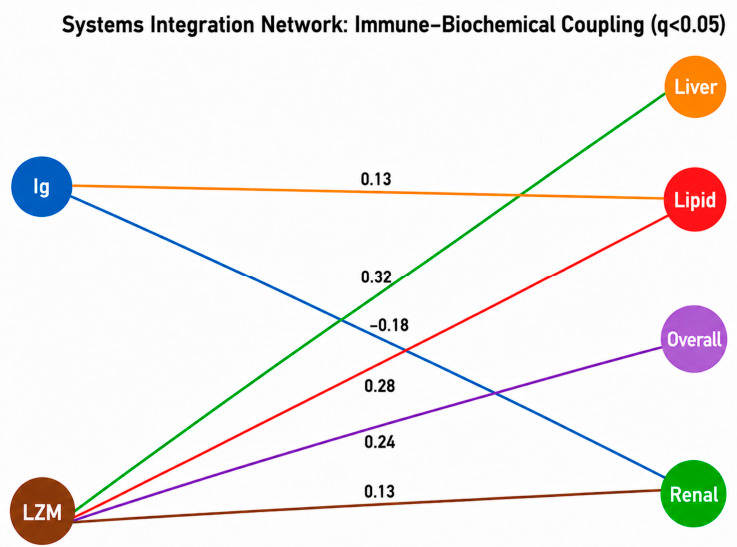
Systems Integration Network of Immune–Biochemical Coupling. Systems integration network showing significant immune–biochemical correlations from within-subject centered Spearman analyses across T0–T5 (*n* = 360). Edges represent FDR-adjusted associations (q < 0.05) with correlation coefficients (ρ).

A systems-level network based on significant within-subject Spearman correlations (FDR-adjusted q < 0.05) integrated immune biomarkers and biochemical indices. Lysozyme emerged as the central node, positively associated with the Liver (ρ = 0.319, q = 6.20 × 10^−9^), Lipid (ρ = 0.277, q = 4.42 × 10^−7^), and Overall Biochemical indices (ρ = 0.242, q = 1.13 × 10^−5^), and negatively with the Renal Index (ρ = −0.134, q = 0.0224). Immunoglobulin showed weaker associations with the Renal (ρ = −0.177, q = 1.81 × 10^−3^) and Lipid indices (ρ = 0.129, q = 0.0237) (Figure 7). No correlations were observed with inflammatory hematological markers, indicating immune activation without systemic inflammation.

Lysozyme showed coordinated coupling with liver, lipid, and overall biochemical indices, with no links to inflammatory hematological markers. This network identifies lysozyme as the key node linking immune activation with metabolic adaptation under stable physiological conditions.

## 4. Discussion

A distinctive feature of the present work is the evaluation of a multi-component immunonutraceutical that combines three natural matrices with complementary immunological properties. Although donkey milk, bovine colostrum, and royal jelly have each been studied individually for their immunomodulatory potential, their combined use within a structured immune-challenge model has rarely been explored [11,12,26,39,40]. Such combined formulations may generate synergistic effects through the interaction of antimicrobial proteins, immunoglobulin-rich fractions, and immunoregulatory lipids [3,6,41].

Donkey milk represented the main component of the formulation and served as an important source of lysozyme. Previous compositional studies indicate that native liquid donkey milk contains lysozyme concentrations approaching 1 g/L, markedly higher than those reported for bovine milk and comparable to human milk [11,14,42]. The biological relevance of dietary lysozyme is supported by experimental evidence showing improvements in immune parameters, antioxidant status, and intestinal microbial balance after supplementation in rabbits [15,38,43]. In addition to its antimicrobial activity, lysozyme is increasingly viewed as a multifunctional innate immune effector that can influence host–microbiota interactions and downstream immune signaling [14,43,44].

Bovine colostrum contributed the immunoglobulin-rich fraction of the formulation. This matrix is known to contain high concentrations of IgG, exceeding 50–70 g/L, together with growth factors and antimicrobial peptides [19]. Clinical studies have shown that bovine colostrum supplementation may improve immune biomarkers and resistance to infection. For instance, Khan et al. described enhanced immune competence in supplemented populations [18].

Royal jelly served as the lipid-derived immunoregulatory component. It contains major royal jelly proteins and bioactive fatty acids such as 10-hydroxy-2-decenoic acid, compounds associated with anti-inflammatory, antimicrobial, and immunomodulatory activities [26,45,46]. Experimental evidence has further shown that royal jelly constituents can modulate immune cell proliferation and cytokine production [47]. Taken together, these three ingredients offer complementary modes of action: antimicrobial proteins from donkey milk, immunoglobulin-rich bioactive compounds from colostrum, and immunoregulatory lipids from royal jelly. This complementarity provided the rationale for combining them in the present study, with the objective of assessing their potential synergistic immunonutritional action.

Within this framework, the present findings show that supplementation with the multi-component formulation promoted coordinated modulation of innate and adaptive immune responses in a controlled rabbit model. The group receiving supplementation before antigen exposure (G4) displayed the strongest humoral response, with an endpoint immunoglobulin concentration of 26.00 ± 5.80 mg/mL, compared with 22.51 ± 8.42 mg/mL in G2 and 17.19 ± 8.46 mg/mL in the vaccination-only group (G1) These results suggest that immunonutritional pre-conditioning may influence systemic humoral immune responsiveness and support immune-related physiological adaptation during antigenic challenge conditions.

Comparable magnitudes of immunoglobulin enhancement have been described following nutritional interventions involving bovine colostrum. Khan et al. reported favorable changes in humoral immune markers together with reduced infection risk in supplemented individuals [18]. Experimental models have also documented increases of roughly 20–30% in circulating antibody concentrations after immunonutritional interventions [3,5,48].

The immunological role of lysozyme is also supported by previous experimental work. Supplementation studies in rabbits and other models have shown improvements in immune markers, antioxidant capacity, and intestinal microbial balance [38,43]. Lysozyme has also been recognized as a natural feed additive capable of shaping host immunity and gut microbial communities [49]. Given its involvement in microbial recognition and early immune signaling, lysozyme may contribute to stronger adaptive activation by facilitating pathogen clearance and antigen processing. The observed association between immunoglobulin and lysozyme was statistically significant but modest in magnitude, indicating limited shared variance between the two biomarkers. From a translational perspective, these findings provide a basis for future investigation of multi-component immunonutritional strategies in the human population, particularly under conditions of increased immunological demand.

Another important outcome of the study is that immune activation was accompanied by coordinated metabolic adjustment. Composite biochemical indices and multivariate analyses revealed structured remodeling across hepatic, lipid, and protein metabolism domains without signs of organ dysfunction. Liver- and renal-related biochemical parameters remained within physiological ranges throughout the study, supporting preserved hepatic and renal integrity. These observations indicate that immune stimulation was associated with adaptive metabolic shifts rather than pathological disturbance. The coordinated early T0→T1 shifts observed across several biochemical and immunological variables likely reflect early physiological adaptation to experimental conditions, repeated handling, and longitudinal sampling procedures.

This interpretation is consistent with the concept of immunometabolism, which describes the bidirectional relationship between immune activity and metabolic regulation [27,28,50]. During immune responses, pathways involved in lipid synthesis, mitochondrial function, and cellular energetics become tightly linked to immune cell activation. The associations identified here between lysozyme dynamics and lipid-related indices align well with this framework. Lipid metabolism plays a central role in membrane remodeling, immune signaling, and inflammatory mediator synthesis [29], and similar links between nutrition, metabolism, and immune function have been described previously [5,6]. Calder et al. emphasized that nutrients and food-derived bioactives can modulate both innate and adaptive immunity through pathways involving lipid mediators, antioxidant defenses, and cytokine signaling, whereas Wu et al. showed that nutritional factors can influence immune cell activation through energy metabolism, mitochondrial regulation, and lipid signaling pathways [3,5]. In the same direction, studies on bovine colostrum and other nutraceutical ingredients have reported immune stimulation in parallel with stable biochemical profiles, suggesting metabolic adaptation rather than systemic toxicity [39,40]. Accordingly, the present results support the view that immune activation took place within a regulated metabolic context [51]. This observation is particularly relevant for the development of functional foods and nutraceuticals intended to support immune health while preserving metabolic homeostasis [52]. In human nutrition, such coordinated immune–metabolic responses may be especially valuable during periods of elevated physiological challenge.

The hematological findings further support the good tolerability of the intervention. Mean leukocyte counts ranged from 6.35 ± 0.54 to 8.19 ± 1.19 K/µL, remaining within development of functional foods and nutraceuticals physiological limits for rabbits. Erythrocyte-related indices were likewise stable, with RBC counts around 5.6–5.8 M/µL and hemoglobin concentrations of approximately 12.6–12.8 g/dL. Altogether, these data indicate that the formulation did not induce hematological abnormalities or evidence of systemic toxicity.

A particularly relevant aspect is the stability of the neutrophil-to-lymphocyte ratio across groups. Because NLR is widely used as an indicator of systemic inflammatory stress and is associated with inflammatory conditions in both experimental and clinical contexts [53], its lack of elevation suggests that immune enhancement occurred without triggering inflammatory disequilibrium. This pattern is in line with previous studies on functional food ingredients. Bovine colostrum supplementation, for example, has been shown to improve immune and mucosal defense markers without inducing adverse hematological inflammatory changes in athletes and physically active populations [39], and broader reviews have emphasized its favorable safety profile [40]. Similarly, royal jelly has been associated with antioxidant and anti-inflammatory effects without adverse hematological consequences [26], while donkey milk and related functional dairy products are generally regarded as well tolerated sources of bioactive proteins that support immune function [11]. Thus, the stable hematological profile observed here is consistent with previous evidence indicating that functional food ingredients can promote immune activity while maintaining inflammatory and hematological equilibrium. For human nutrition, maintaining this balance is particularly important, since effective nutritional support should reinforce host defense without provoking systemic stress. The relevance of these observations for human populations remains to be established through appropriately designed clinical studies.

Although the formulation components investigated in the present study are used in human nutrition, the current findings should be considered hypothesis-generating and interpreted cautiously. Extrapolation from rabbit models to human physiology is inherently limited, and additional clinical studies are required to determine whether comparable immune and metabolic responses occur in humans [17,18,26,54]. Consequently, the present results should be viewed as a basis for future translational research rather than direct evidence supporting nutritional recommendations in humans.

From the standpoint of novelty, this study represents one of the first controlled investigations evaluating a standardized multi-component immunonutraceutical combining donkey milk, bovine colostrum, and royal jelly within a structured immune-challenge model. While these ingredients have been individually investigated for their immunomodulatory properties, their combined administration within a single formulation and under different supplementation schedules relative to antigen exposure has not been previously characterized. A particularly novel aspect of the study is the evaluation of timing-dependent immune effects, including an immunonutritional pre-conditioning strategy prior to antigenic challenge. Furthermore, the integration of longitudinal humoral and innate immune monitoring with biochemical, hematological, and systems-level immune–metabolic analyses provided a broader assessment of physiological adaptation than is typically reported in studies focusing on isolated immune markers.

Several limitations should nevertheless be acknowledged. Although rabbits are well-established models for immunological research, the findings cannot be directly extrapolated to human physiology. In addition, the individual contribution of each formulation component was not assessed separately, making it difficult to determine the relative influence of donkey milk, bovine colostrum, and royal jelly on the overall response. While total serum immunoglobulin concentrations provided useful information regarding systemic humoral immune dynamics, antigen-specific antibody assays were not performed. Vaccination was also administered at different time points in G3 and G4, limiting the ability to fully disentangle supplementation timing effects from natural post-vaccination immune kinetics. Finally, the study focused primarily on systemic immune, biochemical, and hematological responses and therefore does not provide detailed insight into the molecular and cellular mechanisms underlying the observed effects. Future studies employing factorial designs, dose–response approaches, and targeted mechanistic investigations are warranted to further clarify the biological pathways involved.

Despite these limitations, the present findings provide novel evidence that a multi-component immunonutritional strategy can strengthen coordinated immune responses while preserving metabolic and hematological stability. Collectively, these results contribute to the growing body of evidence supporting the potential role of functional foods and immunonutraceutical formulations in the nutritional modulation of immune function.

## 5. Conclusions

This study provides one of the first controlled evaluations of a standardized multi-component immunonutraceutical combining donkey milk, bovine colostrum, and royal jelly within a structured immune-challenge rabbit model. Supplementation modulated both innate and humoral immune biomarkers, with the strongest humoral response observed when the formulation was administered prior to antigen exposure, indicating that immunonutritional pre-conditioning may improve immune responsiveness. Importantly, immune activation occurred alongside stable metabolic and hematological profiles, demonstrating coordinated immunometabolic adaptation without evidence of systemic toxicity or inflammatory imbalance. By integrating immune, biochemical, and hematological analyses within a single experimental framework, the study provides a comprehensive view of how complementary natural bioactive matrices can influence immune–metabolic interactions. Overall, the findings suggest that multi-component immunonutritional strategies may enhance immune readiness while maintaining physiological stability. Although further translational and clinical studies evaluating multi-component immunonutritional strategies in humans are required, these results highlight the potential of functional foods and nutraceutical formulations based on complementary natural ingredients to support immune resilience in human nutrition.

## Figures and Tables

**Table 1:** Ingredients and chemical composition of the commercial diet (% as dry matter basis).

Feed Ingredients	%
Alfalfa meal	24.5
Soybean hulls	19.5
Sunflower meal	14.0
Wheat	12.0
Barley	10.0
Corn	8.0
Wheat bran	6.0
Vegetable oil	1.5
Limestone (CaCO_3_)	1.5
Monocalcium phosphate	1.0
Salt	0.5
L-lysine HCl	0.5
DL-methionine	0.5
Premix	0.5
Total	100
**Chemical composition (% as dry matter basis)**	
Dry matter	90.3
Crude protein	16.5
Ether extract	3.5
Crude fiber	17.5
Ash	7.3
Calcium	1.0
Phosphorus	0.60
Sodium	0.21
Methionine	0.3
Lysine	0.9

**Table 2:** Experimental Groups and Abbreviations.

Group No.	Abbreviation	Full Description	Intervention Scheme
G1	V	Vaccinated only	Vaccination at T0; no IMN supplementation
G2	IMN	Immunonutraceutical only	IMN administered for 6 weeks; no vaccination
G3	V + IMN	Vaccination + Immunonutraceutical	Vaccination at T0; IMN started the day after vaccination and continued for 6 weeks
G4	IMN + V + IMN	Pre-conditioned Immunonutraceutical + Vaccination + Continued IMN	IMN administered from T0–T3; vaccination after T3; IMN resumed the day after vaccination and continued until T5

V = vaccination; IMN = multi-component immunonutraceutical formulation.

## Data Availability

The data supporting the findings of the study are available within the article and Supplementary File.

## Supplementary Materials

The following supporting information can be downloaded at: https://www.mdpi.com/article/10.3390/nu18121872/s1, Table S1. Total serum immunoglobulin (Ig) concentrations by group and time point; Table S2. Serum lysozyme (LZM) concentrations by group and time point; Table S3. Biochemical parameters by group and time; Table S4. Hematological parameters at the final time point (T5).
