# Supplementary material for: Functional Immunonutraceutical Supplementation Enhances Humoral and Innate Immune Dynamics in a Controlled Immune-Challenge Rabbit Model
Source: Nutrients. 2026 Jun 10;18(12):1872. doi: 10.3390/nu18121872 (PMC13305565; doi:10.3390/nu18121872)
Supplement: Supplementary file 1 [file nutrients-18-01872-s001.zip › nutrients-4321434-supplementary.pdf]

Supplementary files

Supplementary Table S1. Total serum immunoglobulin (Ig) concentrations by group and time point

Time point	G1			G2			G3			G4			One-way ANOVA	Kruskal–Wallis
	Mean	SD	SEM	Mean	SD	SEM	Mean	SD	SEM	Mean	SD	SEM	p	p
**T0**	17.190	8.460	2.184	19.345	6.247	1.613	24.186	8.716	2.250	19.586	7.024	1.814	0.097694	0.158252
**T1**	15.138	6.839	1.766	17.073	4.833	1.248	16.277	7.002	1.808	17.069	5.680	1.466	0.802731	0.645524
**T2**	11.384	6.577	1.698	19.358	8.875	2.291	12.969	2.800	0.723	19.856	9.216	2.380	0.002845	0.003198
**T3**	17.026	3.140	0.811	20.567	8.987	2.320	17.340	6.515	1.682	17.355	5.798	1.497	0.397712	0.736334
**T4**	16.576	4.916	1.269	21.267	8.774	2.265	18.979	5.059	1.306	23.597	5.615	1.450	0.022626	0.023843
**T5**	17.385	4.878	1.260	22.511	8.424	2.175	20.539	4.802	1.240	25.995	5.796	1.496	0.003223	0.005624

Notes

Values are means derived from the raw longitudinal dataset (n = 15 animals/group/time point).

SD = standard deviation; SEM = standard error of the mean.

One-way ANOVA and Kruskal–Wallis p-values are shown for each time point.

Supplementary Table S2. Serum lysozyme (LZM) concentrations by group and time point

Time point	G1			G2			G3			G4			One-way ANOVA	Kruskal–Wallis
	Mean	SD	SEM	Mean	SD	SEM	Mean	SD	SEM	Mean	SD	SEM	p	p
**T0**	1.833	0.221	0.057	1.911	0.575	0.149	1.931	0.307	0.079	1.926	0.453	0.117	0.907659	0.819333
**T1**	1.848	0.218	0.056	2.041	0.605	0.156	2.021	0.349	0.090	2.162	0.460	0.119	0.269384	0.235182
**T2**	1.870	0.205	0.053	2.079	0.617	0.159	2.224	0.334	0.086	2.284	0.472	0.122	0.055226	0.036340
**T3**	1.895	0.204	0.053	2.124	0.621	0.160	2.306	0.329	0.085	2.221	0.481	0.124	0.072515	0.029757
**T4**	1.903	0.212	0.055	2.114	0.600	0.155	2.388	0.333	0.086	2.279	0.534	0.138	0.025392	0.008847
**T5**	1.916	0.222	0.057	2.175	0.633	0.163	3.130	0.438	0.113	2.360	0.564	0.146	0.000000	0.000004

Notes

Values are means derived from the raw longitudinal dataset (n = 15 animals/group/time point).

SD = standard deviation; SEM = standard error of the mean.

One-way ANOVA and Kruskal–Wallis p-values are shown for each time point.

Supplementary Table S3. Biochemical parameters by group and time

	1.GPT												
	G1			G2			G3			G4			One-way ANOVA
Time	Mean	SD	SEM	Mean	SD	SEM	Mean	SD	SEM	Mean	SD	SEM	p-value
T0	33.06667	6.902036	1.782098	34.06667	6.238895	1.610876	36.26667	8.713427	2.249797	33.6	6.631096	1.712142	0.634606
T1	29.4	4.239272	1.094575	27.4	4.91063	1.267919	32.73333	10.36799	2.677004	27.8	4.126569	1.065476	0.108037
T2	38.8	10.52344	2.717141	31.2	7.143429	1.844425	39.26667	11.00952	2.842646	30.8	7.866747	2.031185	0.015814
T3	30.4	8.525592	2.201298	32.06667	7.126076	1.839945	28.2	7.350413	1.897868	24.73333	5.444088	1.405658	0.042451
T4	28.26667	5.737927	1.481526	28.8	7.379508	1.905381	33.4	8.033857	2.074333	30.8	6.991832	1.805283	0.195508
T5	27.53333	5.330059	1.376215	27.86667	7.11002	1.835799	27.46667	5.409868	1.396822	28.73333	6.963852	1.798059	0.941944
	2.GOT												
	G1			G2			G3			G4			One-way ANOVA
Time	Mean	SD	SEM	Mean	SD	SEM	Mean	SD	SEM	Mean	SD	SEM	p-value
T0	33.93333	9.69143	2.502316	18.66667	3.77334	0.974272	20.2	2.980892	0.769663	19.2	3.529468	0.911305	2.44E-10
T1	14.53333	2.559762	0.660928	13.06667	1.579632	0.407859	19.13333	7.414914	1.914523	12.73333	1.279881	0.330464	0.000166
T2	20	4.795832	1.238278	14.26667	2.120198	0.547433	19.33333	2.554175	0.659485	17.73333	4.431489	1.144206	0.000313
T3	17.46667	3.50238	0.904311	15.13333	3.226379	0.833048	13.73333	1.869556	0.482717	13.4	2.063284	0.532738	0.000584
T4	15.73333	4.350151	1.123204	14.4	2.02837	0.523723	18.4	3.737837	0.965105	16	2.236068	0.57735	0.012448
T5	15.46667	3.795988	0.98012	13.8	1.656157	0.427618	15.86667	4.189556	1.081739	13.86667	4.257207	1.099206	0.282312
	3.TBIL												
	G1			G2			G3			G4			One-way ANOVA
Time	Mean	SD	SEM	Mean	SD	SEM	Mean	SD	SEM	Mean	SD	SEM	p-value
T0	1.34	0.511021	0.131945	1.04	0.360159	0.092993	0.986667	0.135576	0.035006	1.013333	0.292445	0.075509	
T1	0.646667	0.124595	0.03217	0.626667	0.138701	0.035813	0.62	0.242605	0.06264	0.5	0.084515	0.021822	
T2	0.54	0.098561	0.025448	0.786667	0.354293	0.091478	0.666667	0.183874	0.047476	0.8	0.464451	0.119921	
T3	0.72	0.114642	0.029601	0.886667	0.368136	0.095052	0.573333	0.079881	0.020625	0.586667	0.172654	0.044579	
T4	0.626667	0.143759	0.037118	0.8	0.4326	0.111697	0.9	0.541163	0.139728	0.72	0.137321	0.035456	

T5	0.6	0.106904	0.027603	0.666667	0.313202	0.080868	0.6	0.106904	0.027603	0.653333	0.209989	0.054219	
	**4.CRE**												
	**G1**			**G2**			**G3**			**G4**			One-way ANOVA
Time	Mean	SD	SEM	Mean	SD	SEM	Mean	SD	SEM	Mean	SD	SEM	p-value
T0	0.790667	0.116525	0.030087	0.650667	0.07787	0.020106	0.71	0.089043	0.022991	0.664	0.082358	0.021265	0.000451
T1	0.55	0.048844	0.012611	0.427333	0.077962	0.02013	0.43	0.039461	0.010189	0.446667	0.092014	0.023758	8.57E-06
T2	0.822667	0.156226	0.040337	0.605333	0.213905	0.05523	0.63	0.040356	0.01042	0.592667	0.175233	0.045245	0.000545
T3	0.61	0.087423	0.022573	0.634	0.176222	0.0455	0.55	0.057817	0.014928	0.479333	0.041139	0.010622	0.000717
T4	0.682667	0.144443	0.037295	0.556667	0.193009	0.049835	0.83	0.085189	0.021996	0.746	0.051934	0.013409	4.69E-06
T5	0.63	0.079552	0.02054	0.548	0.188914	0.048777	0.63	0.079552	0.02054	0.676	0.100271	0.02589	0.04204
	**5.ALP**												
	**G1**			**G2**			**G3**			**G4**			One-way ANOVA
Time	Mean	SD	SEM	Mean	SD	SEM	Mean	SD	SEM	Mean	SD	SEM	p-value
T0	510.8667	62.38345	16.10734	517.4667	82.87845	21.39912	544	79.52358	20.5329	515.1333	90.52456	23.37334	0.660538
T1	447.2	46.76109	12.07366	387.2667	78.87827	20.36628	444.4	121.8382	31.45848	385.1333	72.72342	18.77711	0.069306
T2	498.2667	70.17373	18.11878	392.8	45.28828	11.69338	486	82.10533	21.19951	465.8667	123.4544	31.87578	0.005879
T3	385.2	49.67782	12.82676	397.7333	49.88339	12.87984	369.5333	106.9158	27.60553	352.7333	68.85022	17.77705	0.365495
T4	369	52.72029	13.61232	390.4667	107.0666	27.64449	426.7333	67.44889	17.41523	468.8	86.37311	22.30144	0.007747
T5	355.4667	40.16549	10.37069	388.4	101.866	26.30169	353.2667	41.16772	10.62946	384.5333	92.77613	23.95469	0.430731
	**6.TCHO**												
	**G1**			**G2**			**G3**			**G4**			One-way ANOVA
Time	Mean	SD	SEM	Mean	SD	SEM	Mean	SD	SEM	Mean	SD	SEM	p-value
T0	53.73333	9.369149	2.419104	58.26667	27.62108	7.131731	49.73333	8.91601	2.302104	58.73333	20.88563	5.392646	0.505191
T1	58.73333	10.06029	2.597557	54	17.65139	4.557568	54.6	11.98094	3.093465	53.4	18.25924	4.714517	0.757861
T2	57.46667	19.78768	5.109158	42.46667	19.88491	5.134261	42.33333	8.715066	2.25022	41.86667	20.46204	5.283277	0.052817
T3	62.13333	20.98253	5.417667	45.33333	18.7261	4.835057	42.4	11.14707	2.878161	53.86667	22.55427	5.823488	0.025393
T4	60.26667	14.89231	3.845178	38.86667	20.05659	5.178588	59.73333	17.54776	4.530812	86.4	36.64073	9.460595	2.25E-05

T5	63.86667	14.52518	3.750386	36.93333	19.50262	5.035556	64	14.37756	3.712271	57.86667	21.95732	5.669355	0.000216
	**7.HDLC**												
	**G1**			**G2**			**G3**			**G4**			One-way ANOVA
Time	Mean	SD	SEM	Mean	SD	SEM	Mean	SD	SEM	Mean	SD	SEM	p-value
T0	19.46667	5.18055	1.337612	23.06667	4.817626	1.243906	19.86667	3.97971	1.027557	21.86667	5.578872	1.440458	0.161215
T1	20.46667	4.356713	1.124899	20.93333	4.72783	1.220721	19.73333	5.573748	1.439136	21.2	5.821144	1.503013	0.872923
T2	19.53333	6.423914	1.658647	16.73333	5.063407	1.307366	15	4.27618	1.104105	17	5.529144	1.427619	0.156001
T3	23.86667	7.717019	1.992526	17.2	4.857983	1.254326	20.6	6.706074	1.731501	21.26667	5.59932	1.445738	0.046285
T4	24.8	6.537802	1.688053	17.13333	4.688385	1.210536	28.2	7.580049	1.95716	32.53333	6.266312	1.617955	1.52E-07
T5	23.8	6.073361	1.568135	17.86667	4.823553	1.245436	22.13333	7.049485	1.820169	26.13333	5.370111	1.386557	0.002866
	**8.TG**												
	**G1**			**G2**			**G3**			**G4**			One-way ANOVA
Time	Mean	SD	SEM	Mean	SD	SEM	Mean	SD	SEM	Mean	SD	SEM	p-value
T0	93.8	23.28457	6.012051	74.06667	14.52813	3.751148	104	41.02787	10.59335	76.26667	10.84611	2.800453	0.004604
T1	87.2	27.93155	7.211895	73.86667	13.17393	3.401494	107.7333	25.5831	6.605529	75.26667	21.23833	5.483713	0.000378
T2	99	33.11883	8.551246	70.86667	20.02094	5.169385	91.33333	27.98384	7.225396	71.53333	21.10811	5.450091	0.007277
T3	67	24.95138	6.442419	73.13333	15.43589	3.985529	50.13333	16.76249	4.328055	47.26667	14.24513	3.678077	0.000386
T4	57.13333	24.96245	6.445276	68	22.14885	5.718808	63.6	12.36239	3.191954	56.4	14.60333	3.770563	0.3048
T5	60.73333	25.06126	6.470789	67.06667	22.50862	5.811701	61	25.20771	6.508602	50.4	10.02711	2.588988	0.215755
	**9.TP**												
	**G1**			**G2**			**G3**			**G4**			One-way ANOVA
Time	Mean	SD	SEM	Mean	SD	SEM	Mean	SD	SEM	Mean	SD	SEM	p-value
T0	6.193333	0.587326	0.151647	5.646667	0.427395	0.110353	5.7	0.7329	0.189234	5.6	0.356571	0.092066	0.014739
T1	4.84	0.241424	0.062335	4.806667	0.303472	0.078356	4.9	0.335942	0.08674	4.753333	0.315926	0.081572	0.602636
T2	5.8	0.39821	0.102817	5.193333	0.652979	0.168598	5.3	0.320713	0.082808	5.1	0.729971	0.188478	0.004881
T3	5.466667	0.456175	0.117784	5.4	0.656832	0.169593	4.6	0.573212	0.148003	4.7	0.843462	0.217781	0.000294
T4	5.493333	0.678724	0.175246	5.2	0.527799	0.136277	6	0.771825	0.199284	6.193333	0.787824	0.203415	0.000848

T5	5.693333	0.678724	0.175246	5.313333	0.557887	0.144046	5.693333	0.678724	0.175246	5.066667	0.73355	0.189402	0.029943
	**10.ALB**												
	**G1**			**G2**			**G3**			**G4**			One-way ANOVA
Time	Mean	SD	SEM	Mean	SD	SEM	Mean	SD	SEM	Mean	SD	SEM	p-value
T0	5.766667	0.330944	0.085449	5.52	0.461674	0.119204	5.6	0.414039	0.106904	5.506667	0.413118	0.106667	0.286788
T1	4.7	0.392792	0.101419	4.733333	0.465475	0.120185	4.8	0.602376	0.155533	4.7	0.494253	0.127615	0.937944
T2	5.753333	0.304412	0.078599	5.326667	0.312745	0.08075	5.5	0.430946	0.11127	5.5	0.40708	0.105108	0.023338
T3	5.4	0.40708	0.105108	5.386667	0.282506	0.072943	4.4	0.974679	0.251661	5.04	0.703867	0.181738	0.000197
T4	5.6	0.374166	0.096609	5.42	0.391335	0.101042	5.86	0.289828	0.074833	5.186667	1.81063	0.467503	0.273834
T5	5.513333	0.358303	0.092513	5.326667	0.428397	0.110612	5.513333	0.358303	0.092513	5.3	0.501427	0.129468	0.330571

Notes

Values are means from biochemical measurements derived from the raw longitudinal dataset (n = 15 animals/group/time point).

SD = standard deviation; SEM = standard error of the mean.

One-way ANOVA and Kruskal–Wallis p-values are shown for each time point.

Supplementary Table S4. Hematological parameters at the final time point (T5)

Parameter	G1			G2			G3			G4			One-way ANOVA	Kruskal–Wallis
	Mean	SD	SEM	Mean	SD	SEM	Mean	SD	SEM	Mean	SD	SEM	p-value	p-value
Basophils (%)	3.01	0.85	0.219	3.13	0.88	0.227	3.09	0.66	0.170	3.9	0.6	0.154	0.006929	0.017869
Basophils (K/µl)	0.23	0.07	0.018	0.23	0.07	0.018	0.2	0.05	0.012	0.27	0.05	0.012	0.011946	0.01873
Eosinophils (%)	1.51	0.59	0.152	1.47	0.5	0.129	1.59	0.5	0.129	1.3	0.65	0.167	0.548207	0.649272
Eosinophils (K/µl)	0.12	0.04	0.010	0.13	0.01	0.002	0.12	0.04	0.010	0.1	0.08	0.020	0.378211	0.309471
HCT (%)	36.39	2.25	0.580	38.49	1.57	0.405	39.2	2.79	0.720	38.05	0.92	0.237	0.002788	0.027595
HGB (g/dL)	12.81	1.12	0.289	12.6	0.68	0.175	12.77	1.17	0.302	12.65	0.25	0.064	0.899358	0.926754
Lymphocytes (%)	58.02	8.84	2.282	61.01	6.69	1.727	62.3	9.04	2.334	62.89	5.11	1.319	0.309707	0.297553
Lymphocytes (K/µl)	3.45	1.02	0.263	4.77	1.08	0.278	3.93	0.52	0.134	4.09	1.22	0.315	0.006571	0.026517
MCH (pg)	22.08	2.08	0.537	22.21	0.75	0.193	21.82	0.66	0.170	22.51	0.49	0.126	0.455601	0.228848
MCHC (g/dL)	33.49	1.83	0.472	32.9	0.62	0.160	32.6	0.69	0.178	32.97	0.89	0.229	0.194756	0.346822
MCV (fL)	64.33	3.68	0.950	67.15	2.87	0.741	66.72	1.78	0.459	67.9	1	0.258	0.002206	0.009272
Mean platelet volume (MPV) (fL)	6.95	1.07	0.276	7.72	0.43	0.111	7.7	1.05	0.271	7.64	0.33	0.085	0.030007	0.119401
Monocytes (%)	1.58	0.88	0.227	1.66	0.84	0.216	1.5	0.35	0.090	1.57	0.44	0.113	0.933739	0.938446
Monocytes (K/µl)	0.08	0.04	0.010	0.11	0.05	0.012	0.09	0.02	0.005	0.11	0.04	0.010	0.033856	0.0648
NLR	0.74	0.35	0.090	0.56	0.19	0.049	0.62	0.23	0.059	0.57	0.29	0.074	0.233542	0.548141
Neutrophils (%)	31.74	5	1.290	32	5.71	1.474	31.23	9.2	2.375	30.71	5.59	1.443	0.952483	0.947064
Neutrophils (K/µl)	2.29	0.73	0.188	2.51	0.62	0.160	2.4	0.81	0.209	2.21	1.14	0.294	0.779502	0.827922
PLT (K/µL)	422.47	108.11	27.913	552.07	70.08	18.094	557.2	76.27	19.692	528	59.11	15.262	4.94E-05	0.008718
RBC (M/µL)	5.77	0.43	0.111	5.77	0.44	0.113	5.83	0.53	0.136	5.6	0.08	0.020	0.454337	0.788545
RDW (%)	13.14	1.68	0.433	14.5	0.24	0.061	14.28	0.78	0.201	14.87	0.41	0.105	5.28E-05	0.001636
Reticulocytes (%)	5.03	1.53	0.395	4.85	1.46	0.376	4.25	0.32	0.082	5.27	0.77	0.198	0.098872	0.04665
Reticulocytes (K/µl)	263.7	85.55	22.08	275.57	63.83	16.480	244.15	30.37	7.841	285.7	43.11	11.130	0.267992	0.209891
WBC (K/µL)	6.49	0.85	0.219	8.19	1.19	0.307	6.35	0.54	0.139	6.91	2.15	0.555	0.001336	0.001521

Notes

Values are means derived from hematological measurements performed at T5 (n = 15 animals/group).

SD = standard deviation; SEM = standard error of the mean.

Both one-way ANOVA and Kruskal–Wallis p-values are shown for completeness; the appropriate test depends on normality and variance assumptions.
